# How Clinicians Perceive Artificial Intelligence–Assisted Technologies in Diagnostic Decision Making: Mixed Methods Approach

**DOI:** 10.2196/33540

**Published:** 2021-12-16

**Authors:** Hyeyoung Hah, Deana Shevit Goldin

**Affiliations:** 1 Information Systems and Business Analytics College of Business Florida International University Miami, FL United States; 2 Nicole Wertheim College of Nursing & Health Sciences Florida International University Miami, FL United States

**Keywords:** artificial intelligence algorithms, AI, diagnostic capability, virtual care, multilevel modeling, human-AI teaming, natural language understanding

## Abstract

**Background:**

With the rapid development of artificial intelligence (AI) and related technologies, AI algorithms are being embedded into various health information technologies that assist clinicians in clinical decision making.

**Objective:**

This study aimed to explore how clinicians perceive AI assistance in diagnostic decision making and suggest the paths forward for AI-human teaming for clinical decision making in health care.

**Methods:**

This study used a mixed methods approach, utilizing hierarchical linear modeling and sentiment analysis through natural language understanding techniques.

**Results:**

A total of 114 clinicians participated in online simulation surveys in 2020 and 2021. These clinicians studied family medicine and used AI algorithms to aid in patient diagnosis. Their overall sentiment toward AI-assisted diagnosis was positive and comparable with diagnoses made without the assistance of AI. However, AI-guided decision making was not congruent with the way clinicians typically made decisions in diagnosing illnesses. In a quantitative survey, clinicians reported perceiving current AI assistance as not likely to enhance diagnostic capability and negatively influenced their overall performance (β=–0.421, *P*=.02). Instead, clinicians’ diagnostic capabilities tended to be associated with well-known parameters, such as education, age, and daily habit of technology use on social media platforms.

**Conclusions:**

This study elucidated clinicians’ current perceptions and sentiments toward AI-enabled diagnosis. Although the sentiment was positive, the current form of AI assistance may not be linked with efficient decision making, as AI algorithms are not well aligned with subjective human reasoning in clinical diagnosis. Developers and policy makers in health could gather behavioral data from clinicians in various disciplines to help align AI algorithms with the unique subjective patterns of reasoning that humans employ in clinical diagnosis.

## Introduction

### Overview

Artificial intelligence (AI) and related technologies are rapidly evolving as part of workplace technology to support clinicians’ decision making [1,2]. AI refers to the use of a collection of intelligent technologies in “the science and engineering of intelligent machines and computational part of the ability to achieve goals in the world” [3] or to “model intelligent behavior with minimal human intervention” [4-6]. Its influence has permeated retail, marketing, and human resource management [6]. Specifically in health care, considering the risks of incorrect predictions, the Consumer Technology Association (CTA) has reconceptualized health care AI as “assistive intelligence,” described as “a category of AI-enabled software that ‘informs’ or ‘drives’ diagnosis or clinical management of a patient,” in which “the health care provider makes the ultimate decisions before clinical action is taken” [7]. In both clinical and administrative areas of health care organizations, AI algorithms are increasingly embedded in health information technologies (HITs) to assist with clinical functions such as data monitoring, clinical research, diagnostics, and support compliance for billing and administrative tasks [8,9]. AI technology in health care is therefore expected to streamline clinicians’ clinical and administrative decision making by providing prompt data analyses and the necessary recommendations to make health care more efficient and cost-effective.

Particularly within the clinical side of health care organizations, clinical decision making is based on subjective and objective patient information and other influencing variables. Clinical decision making generally refers to the process of making a choice among options aimed at diagnosis, intervention, interaction, and evaluation within a context, in which numerous interactions exist among stakeholders, background knowledge, and social and technological factors [10]. In other words, clinicians’ decision making regarding patient health outcomes or health care diagnoses is largely influenced by numerous factors such as the idiosyncratic characteristics of patients and clinicians, consultation environments, and technology used [11]. In this information-rich context, the ability to access and manage appropriate and accurate information is critical for clinicians to make decisions on the patient’s behalf. As reflected in the CTA definition, AI algorithms under these circumstances need to complement human cognitive processing of electronic medical records, multimedia images, or laboratory results. Once successfully incorporated, AI assistance has been shown to help clinicians reduce adverse events outside of the intensive care unit by 44% [12].

Thus far, views have been mixed on how AI may “assist” or “team up with” clinicians through the medical assessment and diagnosis processes. On the one hand, AI allows clinicians to freely follow their own paths of inquiry through patient investigations and select from a database of questions on patient history, physical examinations, and laboratory tests to make patient-specific diagnostic decisions [13]. On the other hand, training AI with a collection of data from the clinicians’ own subjective diagnoses of various patient cases may not be ideal for fully utilizing AI techniques [14]. Indeed, inconsistent performance has also been reported [15]. Given that 83% of health care organizations have a strategy for AI investment and deployment in the coming years [16], particularly for electronic health record management and diagnosis [17], health care practice and training will follow such trends. However, as core users of AI, incumbent health care stakeholders in the current health care system have differential levels of technological readiness; therefore, clinicians’ existing ability to incorporate analytic results from AI may hamper the leveraging of the full potential of AI. In other words, care providers’ attitudes toward and positive experiences with the assistance offered by AI-enabled technologies in the process of clinical diagnosis can help health care organizations reap the benefits of AI investments by enhancing consistency, quality of care, and reducing costs [18].

However, research on whether and how clinicians assess AI assistance in decision-making processes remains limited. Thus far, in health care AI literature, clinicians’ clinical task performance is known to be influenced by clinical task types and evidence-based standards that support AI’s data structure and clinical integration capacity within health care institutions [19]. One stream of AI literature, which has focused on the types of tasks and medical diagnosis, claims that AI algorithms enhance human clinicians’ capabilities while improving efficiency in incident reporting and reducing adverse events [20,21]. However, the quality of health care diagnostic tasks between clinicians and AI continues to be characterized by broad discrepancies [22-24]. According to literature on AI’s data structure, health information management professionals’ ability to improve coded data quality and data patterns is necessary to ensure the optimal adaptation of teaming with AI [25]. Moreover, per the literature on the clinical integration of AI, clinicians’ perceptions should be recognized to enhance the clinical diagnosis by AI [21,26-29].

In summary, our literature review revealed that, although advanced AI algorithms have the potential to enhance the quality and efficiency of health care, the critical users of AI algorithms are clinicians whose roles are to understand and communicate with AI. Nonetheless, there has been limited focus in AI health care research on clinicians’ attitudes toward AI assistance in actual diagnostic decision making, which may be due to the infancy of such AI-assisted tools, lack of trust in AI from health care stakeholders, and potential health-related risks. Therefore, there is reasonable urgency to examine clinicians’ attitudes and sentiments toward AI assistance in clinical decision making, to address the gaps in the existing body of knowledge.

### Background and Theory

#### Medical Diagnostic Knowledge Theory

Clinicians’ decision making includes diagnoses or high-complexity decisions across medical specialties. A diagnosis is viewed as an iterative process of “task categorization” by decomposing patients’ health symptoms into different task classes and matching the given conditions with the predefined disease categories based on their respective hypotheses [30]. Clinicians are expected to predict and determine the course of action based on their knowledge and experience and by utilizing information on the features of the focal clinical situations. The theory of clinical diagnosis revolves around 2 important concepts: clinical knowledge and clinical reasoning. The former refers to the fundamental base of knowledge, and it can be subdivided into 3 categories: conceptual, strategic, and conditional knowledge [31]. The latter refers to a holistic process of hypothesis generation, pattern recognition, context formulation, diagnostic test interpretation, differential diagnosis, and diagnostic verification, all of which provide both the language and methods of clinical problem solving [31]. Once clinicians have obtained clinical knowledge, they then organize it using mental representations, medical scripts, or clinical examples, followed by cognitive processes to translate medical information into testable hypotheses in each context and evaluate their own clinical reasoning for clinical diagnosis [32]. Specifically, the conceptual framework of a clinical diagnosis includes the “hypothetico-deductive model” (ie, generation of multiple competing hypotheses from preliminary information provided by patients), decision analysis, pattern recognition, and intuition [33]. During this process, challenges exist as to how clinicians match patients’ cases with known patterns and focus on complex patient information among many treatment options [33]. In summary, a typical clinical decision-making process consists of the speedy processing of the situational features, assessment of the relevant hypotheses, and investigations and treatments in a sequence [30].

Such clinical reasoning by individual clinicians cannot be fully supported by AI algorithms’ generalized task categorization, pattern recognition, and matching [34]. The current level of AI performs well for clinical knowledge creation by simple interpretations of medical images, slides, and graphs, as well as detection of complex relational time-series patterns within data sets [35,36]. AI algorithms for clinical reasoning (ie, IBM Watson, chatbots to smartphone apps) are believed to function as decision support tools for making diagnoses, providing patient consultation, and detecting certain medical symptoms [26].

In his book *Deep Medicine*, Eric Topol [37] highlighted the role of AI in clinical diagnosis as augmented intelligence: Some clinical knowledge formation via feature selection, task categorization, and pattern recognition can be aided by AI for clinicians to perform clinical reasoning by making a clinical diagnosis. A clinical diagnosis is a high-complexity decision that needs to be based on information inquiry using idiosyncratic patient data and threshold-based decision rules. Without clearcut threshold rules for decisions, AI may not fully process and analyze idiosyncratic patient cases and inaccurate descriptions from patients; it may likewise fail to incorporate the complexity of the diagnosis and predict patients’ adverse conditions [35]. Therefore, the role of AI in clinical reasoning or clinical diagnosis is expected to be more assistive, and clinicians should communicate well with AI to prevent any adverse effects of clinical reasoning on patients’ health outcomes.

#### Artificial Intelligence Technology Use in Practice and in Simulation

In practice and in simulation, clinicians are users or collaborators of such “assistive” AI, contingent upon the extent and scope of such technologies that are defined within the context. In other words, AI can refer to algorithms that are embedded in existing HITs or holistic technological artifacts to be newly implemented as standalone software. As such, the use of AI is no longer limited to specific HITs in practice. Clinicians may have unintentionally encountered various AI technologies through system interfaces and embedded AI decision-making logic within systems used in medicine. Owing to such mixed definitions of AI in health care [38], the effects of AI-assisted diagnostic performance have been mixed in prior studies. Positive performance is expected when clinicians trust and expect the performance of AI [21], whereas information overload driven by AI algorithms can cause a loss of situational awareness, thereby negatively affecting clinicians’ decision-making abilities [35]. In addition, clinicians are perceived as more trusted by patients [26] and that AI algorithms may not be effective in diagnosing unique cases. Thus, clinicians need to diagnose and communicate with patients using AI, at least for a while. The manner through which clinicians make clinical diagnoses with the support of AI technology and perceive such a diagnostic decision merits further investigation.

Unlike these practical constraints, observations and evaluations of clinicians’ health care diagnostic behaviors have been methods of inducing behavioral changes in safer simulation contexts. As what is learned throughout clinical health care training has been associated with what is likely to be carried out in clinical practice [35] and similar technologies span across practice and simulation contexts [39], clinicians’ AI attitudes and performance have been studied and predicted from their use of simulated AI [40]. Thus, one can examine the way clinicians have used and familiarized themselves with AI in simulation and extrapolate their behaviors in practice.

Given that clinicians’ behaviors and readiness to use this technology can be predicted from simulation experiences [39], we turned to a context of clinical diagnosis simulations—whereby clinicians have familiarized themselves with AI—and evaluated their decisions in a safe and controlled context. Subsequently, we examined whether and how clinicians perceive AI assistance and how their perceptions may differ from other non-AI-based diagnostic situations.

Taken together, in simulation contexts, this study explored users’ detailed experiences with AI-enabled patient diagnosis and examined the effect of AI assistance on diagnostic performance. Thus, the following research questions were formulated to shed light on clinicians’ attitudes and behavioral characteristics regarding AI assistance in patient diagnosis:

- Research question 1. During patient diagnosis, what are clinicians’ sentiments toward AI assistance, and how do their sentiments differ from other non-AI-based diagnostic situations?
- Research question 2. How does current AI assistance affect clinicians’ perceptions of enhancing diagnosis and future care task performance?

## Methods

### Survey Procedure

To this end, our target population was clinicians who have experience with patient diagnosis encounters using AI-based diagnostic technology to understand clinicians’ perceptions of AI-assisted diagnosis in a controlled and safe context where patient care was not compromised by potentially incorrect AI algorithms. We recruited clinicians who met the abovementioned requirement and used AI-based diagnostic technology using live patient simulations in a nursing simulation lab. We accessed 3 cohorts of family nurse practitioner (FNP) students who experienced 3 patient diagnosis simulations in the lab: encounters with live standard patients, encounters with AI assistance in diagnosing patients using multimedia patient information, and encounters with patient simulators (ie, lifelike mannequin patients). Each qualified participant was then incentivized by US $5 Amazon gift cards for their completion and the quality of their responses. Consequently, 144 clinicians were selected and invited to participate in the online simulation surveys.

In the simulation lab at the College of Nursing, AI-enabled diagnosis technology has been used in on-ground lessons, in which faculty and students collaborate to complete virtual patient cases and in home-based diagnostic decision making [13,41,42]. In our university, after an AI system developer implemented the focal AI technology and trained the graduate nursing faculty on the technology and after the successful go-live events, this technology was subsequently integrated as part of the clinical simulations for the students. The software is based on data from hundreds of actual patients compiled by experts and AI and based on physiology algorithms [13]. The use of this system is particularly emphasized in the final year of the program to promote skills building and practice in clinical diagnosis.

This interactive AI incorporates intelligence from both humans and AI physiology algorithms such that evaluators on the other end of the system can recognize any patterns demonstrated by those specific users in the process of clinical diagnosis. Clinicians are known to experience some technical features of AI, such as search ability, knowledge expression function, reasoning ability, abstraction ability, speech recognition ability, and ability to process fuzzy clinical information [13]. In our context, reasoning ability was embedded by FNP faculty in the AI-enabled diagnosis technology so that the participants could make diagnostic decisions with AI assistance and obtain feedback after completing each patient case.

### Survey Instruments

Our online simulation surveys consisted of 2 parts: survey instruments and open-ended simulation questions. First, for the survey instruments, each clinician reported their perception of AI assistance and perceived performance of patient diagnosis and overall clinical tasks using a 7-point Likert scale ranging from “strongly disagree” (1) to “strongly agree” (7). Table 1 presents the key survey items sourced from existing information systems (IS) literature.

As shown in Table 1, our study had 2 dependent variables. One was diagnostic performance, which we defined as the ability to provide health care consultations and diagnose health-related issues properly both in person and via online or telehealth platforms [3]. We contextualized and operationalized clinicians’ diagnostic capabilities in a virtual context (mean 5.39, SD 0.13) [30]. The other dependent variable was clinical task performance, the survey items for which were adapted from the IS literature (mean 5.48, SD 0.12) [43].

We included control variables that accounted for personal technological traits and demographics. On the one hand, personal technological traits were measured via individual levels of technological advancement, such as personal innovativeness [44], technological habits [45], and computer literacy [46]. In the IS literature, personal innovativeness and computer literacy have been salient in explaining the technology adoption behaviors of individual users. Here, we defined personal innovativeness as “the willingness of an individual to try out any new information technology” [1], whereas computer literacy is “a judgement of clinicians’ capability to use a computer” [6]. We also included technology habits to control for the participants’ potential automatic reactions to the use of AI-enabled diagnosis technology that includes multimedia information and similar AI algorithms on social media platforms [47]. Lastly, participants’ demographic characteristics, such as self-identified gender, age, income, education, and occupation, were included in the survey to control for potential confounding effects.

Next, in the part listing open-ended simulation questions, each clinician was asked to describe their experience with patient diagnosis under 3 different diagnostic modalities: diagnosing a live patient, diagnosing a human-like mannequin, and AI-based diagnostic simulations using AI assistance. In the 3 simulation prompts, participants were asked to recall their completed patient cases during the semester and write comments using either keywords or key phrases. For example, particularly for the AI case, the scenario prompt reads as given in Textbox 1. After reading this prompt, participants described “favorite” as well as “least favorite” diagnosis experiences using keywords or key phrases in 2 open-ended questions. Each clinician’s 6 diagnostic encounters were recorded as textual narratives, along with the 3 different simulation contexts of the patient diagnoses.

**Table 1:** Key survey items.

Variables		Survey items^a^	References
**Dependent variables**			
	Diagnostic performance	OO^b^ system allows me to carefully evaluate the health condition of the patient. OO system allows me to thoroughly assess the health condition of the patient. OO system allows me to accurately evaluate the patient’s health condition. OO system allows me to think critically during the simulation experience.	[30]
	Clinical task performance	I believe that the use of OO system can increase my overall performance. I believe that the use of OO system can increase my effectiveness with the care tasks when working with live patients in the future. With the use of OO system, I believe I can work more efficiently for managing care tasks when working with live patients in the future. I believe that the use of OO system can increase the quality of care. I believe that the use of OO system can decrease error rates in communication and information sharing with other care members in the future. I believe that the use of OO system will help me understand what I have learned.	[43]
**Independent variable**			
	AI^c^ assistance	Clinicians’ experience of AI-assisted diagnostic simulation (binary: 1, with AI assistance; 0, live patient encounter with no AI assistance)	
**Key control variables**			
	Personal technology trait: technology habit	The use of social media has become a habit for me at work. I am addicted to using social media at work. I must use social media at work. Using social media has become natural to me at work	[44]
	Personal technology trait: personal innovativeness	If I heard about a new information technology, I would look for ways to experiment with it. In general, I am hesitant to try out new information technologies. Among my peers, I am usually the first to try out new information technologies. I like to experiment with new information technologies.	[45]
	Personal technology trait: computer literacy	I could complete the health care task using health information technology if there was no one around to tell me what to do as I go I could complete the health care task using health information technology if I had just the built-in help menu for assistance. I could complete the health care task using health information technology if someone showed me how to do it first. I could complete the health care task using health information if I had used similar apps before this one to do the same job.	[46]

^a^Each item uses a seven-point Likert scale ranging from “strongly disagree” (1) to “strongly agree” (7).

^b^OO system refers to artificial intelligence–enabled diagnosis technology in our research setting.

^c^AI: artificial intelligence.

**Example scenario of artificial intelligence (AI)–based patient diagnosis.**

You read a case description about a 50-year-old patient on an AI-based diagnosis system. His complaints are about back pain. “My back has been hurting for around five days. I bent over to pick something up in my print shop and I had this severe pain in my back. It hurts so much so it is hard to stand up. The pain is on and off throughout the day—maybe four times, for half an hour each, especially when I am walking around. It is mostly a dull aching pain.”

### Study Design

Using clinicians’ quantitative and qualitative responses from our online simulation surveys, we utilized a mixed method to analyze our mixed data. According to Creswell [48] and Creswell and Clark [49], mixed methods research “incorporates qualitative and quantitative data to solve complex research questions and hypotheses, and it is suitable for explaining what (ie, estimating overall trends in participant behaviors within the population) and obtaining an in-depth understanding of why (ie, specific individual behaviors in the subsamples).” This method is particularly useful for exploring context-specific variables and understanding beyond the importance of research variables by delving into the underlying mechanisms using causal models.

Mixed methods research includes sequential and concurrent designs such that qualitative and quantitative data can be collected sequentially in the former, whereas both types of data are obtained at the same time in the latter [50]. Each category has a specific design typology based on the emphasis on the importance of qualitative or quantitative data, the analysis process, and theoretical emphasis (see Castro et al [50] for an in-depth discussion on designs of mixed methods research). We applied a concurrent triangulation design, because both quantitative and qualitative data were collected concurrently, and such data were used to accurately describe and examine clinicians’ experiences and sentiments toward AI assistance in clinical decision making [50].

### Statistical Analysis

We utilized qualitative and quantitative data using natural language understanding (NLU) and hierarchical linear modeling (HLM). Regarding the first research question—capturing clinicians’ respective sentiments in 3 cases of patient diagnosis simulations—we analyzed textual narratives to understand what clinicians perceived about the diagnosis process in teaming with AI and compared it with non-AI-involved diagnostic situations using NLU. Textual comments consisted of a single sentence or a small number of keywords. To incorporate such data characteristics, we deemed NLU, as a subfield of computer science, an adequate method based on its explicit focus on the use of computational techniques to learn, understand, and produce human language content, and many information technology firms, such as Microsoft, Google, and IBM, have developed NLU platforms and algorithms [51,52]. We utilized IBM Watson Natural Language Understanding-77 for its well-known performance and cross-evaluation results. Understanding each participant’s language content was more suitable than traditional text mining analytics [52-54].

Next, for quantitative data analysis, we implemented HLM estimation techniques to answer the second research question—measuring the effect of the current form of AI assistance on clinicians’ diagnostic decision making and care task performance. In our data, each clinician’s clinical diagnosis experience was nested within the program. In other words, under the same graduate program, each participant was exposed to the same sets of patient diagnosis simulations with 3 modalities and reported their responses with 3 diagnosis simulations repeatedly. This context could engender statistical dependency among the responses [55]. Moreover, in the research model, the outcome variable (ie, the clinical diagnosis) was at the individual level, and the AI assistance variable was at the group level (or graduate nursing program), leading to a multilevel research program in the same research model [21]. To mitigate such dependency and estimate this multilevel model, we used the STATA 16.1 (StataCorp, College Station, TX) xtmixed procedure to carry out generalized linear modeling using the restricted maximum likelihood or residual maximum likelihood estimation. The xtmixed procedure fits linear mixed models that include fixed (or standard regression coefficients) and random effects that are not estimated directly but by variance component. The default covariance structure is independent covariance. We also specified other covariance structures (eg, exchangeable and unstructured) to validate our mixed model results (see [56] for an in-depth discussion of mixed models and covariance structures).

## Results

We explored clinicians’ perceptions and performance prospects toward AI assistance in patient diagnosis research. These relations and sentiments are evidenced by the results of generalized linear models as well as qualitative text analysis with direct quotations from the health care worker sample.

### User Statistics

A total of 114 clinicians completed our online surveys during the 2020-2021 study period (response rate: 114/144, 79.2%). In summary, 66.7% (76/114) of the participants were between 26 years old and 40 years old, 49.1% (56/114) were white, and 84.2% (96/114) identified as female. Additionally, 89.5% (102/114) worked either full time or part time in hospitals or clinics. In terms of education, 24.6% (28/114) had a graduate degree, whereas all the participants had obtained a bachelor’s degree in nursing with prior clinical experience before joining the graduate program, as shown in Table 2.

**Table 2:** Respondent demographics (N=114).

Characteristics			Results, n (%)
**Self-identified gender**			
	Male	16 (14.0)	
	Female	96 (84.2)	
	Not disclosed	2 (1.8)	
**Age (years)**			
	18-25	10 (8.8)	
	26-40	76 (66.7)	
	41-55	26 (22.8)	
	56-65	2 (1.8)	
**Income (US $)**			
	25,000-49,999	20 (17.5)	
	50,000-74,999	32 (28.1)	
	75,000-99,999	30 (26.3)	
	≥100,000	4 (3.5)	
	Prefer not to answer	28 (24.6)	
**Education**			
	Bachelor’s degree	82 (71.9)	
	Master's degree	20 (17.5)	
	PhD	8 (7.0)	
	Others	4 (3.5)	
**Occupation**			
	Working full time	54 (47.4)	
	Working part time	48 (42.1)	
	Unemployed	6 (5.3)	
	Other	6 (5.3)	
**Race**			
	African American	22 (19.3)	
	Asian	8 (7.0)	
	Native Hawaiian or Pacific Islander	2 (1.8)	
	White	56 (49.1)	
	Other	20 (17.5)	
	Prefer not to answer	6 (5.3)	
**Urban/rural area**			
	Urban	98 (86.0)	
	Rural	10 (8.8)	
	Other	6 (5.3)	

### Research Question 1: Sentiment Analysis Results

To identify clinicians’ sentiments toward AI assistance, we compared the clinicians’ perceptions regarding AI-assisted diagnosis and non-AI diagnosis contexts. Table 3 presents some narrative examples in our data set whereby the clinicians described what they liked and disliked about the patient diagnosis process with the help of AI-aided technology, with a live human patient, and with a human-like mannequin/patient simulator.

The results of the NLU analyses are reported in Tables 4, 5, and 6. We found that clinicians perceived their patient diagnosis experience differently across the 3 simulation cases. The positive sentiment scores for the diagnosis process were 0.99 for the live patient, 0.92 for teaming up with AI assistance, and 0.41 for the patient simulator. In contrast, the negative sentiment score was the highest with the patient simulator (sentiment score = –1), followed by the live patient (sentiment score = –0.97) and AI assistance (sentiment score = –0.87). Specifically, our respondents perceived diagnostic simulations with AI technology less negatively and more positively compared with diagnosing a live human patient. Table 4 reports each positive and negative sentiment score for all 3 cases, with scores ranging from –1 to 1.

Tables 5 and 6 present the most relevant keywords from the textual narratives for the 3 simulation cases. The respondents perceived AI-based diagnostic simulations positively for the following reasons: “convenient access,” “thorough assessment skills,” “student interaction convenience,” “interactive learning rationale,” “good learning opportunities” and “[a] vast range of questions.” At the same time, they also perceived the AI-based diagnosis simulation to contain a “long system,” “strict sensitive clicking,” “differential diagnosis,” “large learning curve,” and “technical difficulties,” and they found it to be a “hard system.” These keywords indicated that the current form of AI assistance may lead to differential diagnosis logic relative to clinicians’ own logic and hypotheses, and technical difficulties could cause users to be averse to the technology.

**Table 3:** Some narrative examples in our data set, based on 65 recorded instances.

Narrative valence	AI^a^ assistance context	Non-AI assistance context	
	Diagnosis experience with AI assistance	Diagnosis experience with live patients	Diagnosis experience with HPS^b^
Positive comments	“I don’t feel like I am under pressure and can do it at my own pace”	“interaction, being able to gauge the patient, reading facial expressions, immediate feedback”	“Very realistic, lifelike scenarios”
Negative comments	“not having an orientation on how to work the system (first time user)”	“I'm not an actor, and it felt like acting; immediate feedback”	“being watched through a one-way mirror”

^a^AI: artificial intelligence.

^b^HPS: human patient simulator. It is worth nothing that the clinicians recorded their retrospective experience with the HPS, as it was not used in the graduate program.

**Table 4:** Clinicians’ emotions with patient diagnosis under 3 different scenarios, based on 65 recorded instances.

Sentiment	AI^a^ assistance context	Non-AI assistance context	
	Diagnosis experience with AI assistance	Diagnosis experience with live patients	Diagnosis experience with HPS^b^
Positive sentiment^c^	0.92	0.99	0.41
Negative sentiment^c^	–0.87	–0.97	–1

^a^AI: artificial intelligence.

^b^HPS: human patient simulator. It is worth nothing that the clinicians recorded their retrospective experience with the HPS, as it was not used in the graduate program.

^c^The sentiment score ranged from –1 (negative) to 1 (positive).

**Table 5:** Extracted keywords from clinicians’ positive textual narratives, based on 65 recorded instances.

Keyword		Relevance^a^
**AI^b^ assistance context: diagnosis experience with AI assistance**		
	Convenient access	0.976987
	Thorough assessment skills	0.641589
	Good practice student interaction convenience	0.62027
	Interactive learning rationales	0.612274
	Good learning opportunities	0.599706
	Questions	0.559626
	Vast list of questions	0.542493
	Question banks	0.537307
	Times	0.534484
	Issues	0.518757
	Convenience	0.515202
	Scenario	0.514838
	History	0.513546
	Patient data	0.512584
	Plan	0.507775
	Pressure	0.5077
	Diagnoses	0.507442
	Students	0.507159
	Ease	0.506215
	Choices	0.505864
**Non-AI assistance context: diagnosis experience with live patients**		
	Fast convenient real experience	0.89935
	Fast convenience	0.706168
	Real-life situation	0.68436
	High quality	0.68049
	Telehealth: convenient fast access	0.664928
	Real world	0.602068
	Real life	0.600372
	Live patient	0.581455
	Physical actions convenience	0.581031
	Video interactions	0.561663
	Convenience	0.546701
	Best learning experience	0.540142
	Challenging open-ended questions	0.536408
	Live experience	0.534688
	Positive feedback	0.533209
	Patients	0.532791
	Facial expressions	0.529643
	Experience	0.528151
	Common complaint	0.526369
**Non-AI assistance context: diagnosis experience with HPS^c^**		
	Good practice	0.721493
	Real patient	0.696931
	Less fear	0.695137
	Convenient reinforcement of learning	0.633235
	Better interaction	0.60778
	Real world	0.591024
	New things	0.58642
	Future trend	0.58135
	Clear case	0.581078
	Convenience	0.554921
	Patient simulators	0.543254
	Point	0.527845
	Scenarios	0.519927
	Mistake	0.518499
	Practice maneuvers	0.513818
	Patients	0.512968
	Experience	0.512967
	Ease	0.510172
	Survey	0.507779
	Person	0.507779

^a^Relevance scores range from 0 to 1, reflecting that higher values indicate greater relevance.

^b^AI: artificial intelligence.

^c^HPS: human patient simulator. It is worth nothing that the clinicians recorded their retrospective experience with the HPS, as it was not used in the graduate program.

**Table 6:** Extracted keywords from clinicians’ negative textual narratives, based on 65 recorded instances.

Keyword		Relevance^a^
**AI^b^ assistance context: diagnosis experience with AI assistance**		
	First-time user	0.837534
	Long system	0.779516
	Strict sensitive clicking	0.634627
	Differential diagnosis	0.62574
	Large learning curve	0.610383
	Technical difficulties	0.584259
	Hard system	0.573913
	Results of x-rays and CT^c^	0.567441
	Sound doesn’t work	0.552959
	Cases	0.545605
	Next part	0.542253
	Complex	0.538614
	Times	0.531118
	Area	0.522729
	Orientation	0.520495
	List	0.518181
	Real patient	0.513188
	User	0.512912
	Work	0.510401
	Things	0.509941
**Non-AI assistance context: diagnosis experience with live patients**		
	Strict testing environment	0.7036
	Face interaction complex	0.65795
	Limited time	0.65711
	Short time	0.60814
	Constant need	0.59631
	Real clinic patients	0.59044
	Physical examination (PE)	0.5563
	Sound effect	0.55628
	Feeling of self-doubt	0.54479
	Accurate data	0.54182
	Assess patient	0.5376
	Patient expresses lack of physical exam	0.53226
	Feedback	0.53139
	Interaction	0.52951
	Actor	0.5278
	Minutes	0.51986
	PE doesn’t correlate	0.51702
	Quality distractions	0.51557
	Unreliability	0.51449
	Client	0.51445
**Non-AI assistance context: diagnosis experience with HPS^d^**		
	Human experience	0.71766
	Physical examination maneuvers	0.65952
	Lack of feelings response	0.55535
	Live patient additional questions	0.5476
	Lab values	0.53833
	Unrealistic prefer	0.53269
	Actual patient experiences	0.52713
	Expressions	0.52625
	Immediate feedback	0.51753
	HPS encounters	0.51753
	Scenario	0.51542
	Simulators	0.51339
	Student	0.51188
	Real patient	0.51112
	Assessment	0.50641
	Reaction	0.50641
	Survey	0.50641
	Realistic interaction	0.49813
	Sounds effects	0.48723
	One-way mirror	0.48654

^a^Relevance scores range from 0 to 1, reflecting that higher values indicate greater relevance.

^b^AI: artificial intelligence.

^c^CT: computed tomography.

^d^HPS: human patient simulator.

### Quantitative Methods: Results of Mixed Models

Table 7 presents our findings for the second research question—the effect of AI assistance on clinicians’ clinical diagnosis and care performance from multilevel mixed effects models. Models 1 and 2 report the results for the 2 dependent variables of diagnostic performance and clinical task performance, respectively, where the independent variable is the experience with AI assistance (binary). In terms of individual-level covariates, social media use, personal innovativeness, and computer literacy were included as personal technological traits, in addition to demographic covariates. The HLM results were compared with the baseline ordinary least squares model with clustered standard errors. The effect of AI assistance was not statistically significant in explaining the variation in enhanced clinical diagnosis. Instead, education and age, which were related to clinicians’ overall practical experience, were positively associated with clinical diagnosis. The mixed model results were qualitatively identical, along with the different covariance structures.

**Table 7:** Results from hierarchical linear modeling (N=114 observations).

Variables		Model 1^a^					Model 2^b^			
		OLS^c^ (clustered SE^d,^^e^)	*P* values	Mixed model^f,g^	*P* values	OLS (clustered SE^d,h^)		*P* values	Mixed model^f,g^	*P* values
Constant		2.162 (1.013)	.04	2.162 (1.851)	.24	4.278 (0.898)		<.001	4.278 (1.462)	.003
AI assistance		–0.105 (0.185)	.57	–0.105 (0.167)	.53	–0.421 (0.192)		.03	–0.421 (0.175)	.02
**Personal technological traits**										
	Technology habit	0.232 (0.104)	.03	0.232 (0.137)	.09	0.244 (0.0803)		.004	0.244 (0.108)	.02
	Personal innovativeness	–0.227 (0.202)	.27	–0.227 (0.197)	.25	–0.0234 (0.157)		.88	–0.0234 (0.155)	.89
	Computer literacy	–0.161 (0.181)	.38	–0.161 (0.157)	.31	–0.257 (0.111)		.02	–0.257 (0.124)	.04
**Control variables**										
	Female gender	–0.202 (0.615)	.74	–0.202 (0.575)	.73	0.0792 (0.495)		.87	0.0792 (0.454)	.86
	Age: 18-25 years	1.885 (0.892)	.04	1.885 (1.473)	.20	–0.910 (0.825)		.28	–0.910 (1.163)	.43
	Age: 26-40 years	2.339 (0.782)	.004	2.339 (1.294)	.07	–0.236 (0.704_		.74	–0.236 (1.021)	.82
	Age: 41-55 years	2.428 (0.802)	.004	2.428 (1.321)	.07	0.102 (0.683)		.88	0.102 (1.042)	.92
	Race: African American	–0.0232 (0.540)	.97	–0.0232 (0.842)	.98	0.00592 (0.615)		.99	0.00592 (0.664)	.99
	Race: Asian	–0.419 (0.687)	.55	–0.419 (1.106)	.71	–0.0125 (0.745)		.99	–0.0125 (0.873)	.99
	Race: Native Hawaiian/Pacific Islander	1.067 (0.672)	.12	1.067 (1.514)	.48	0.708 (0.724)		.33	0.708 (1.195)	.55
	Race: White	–0.0132 (0.417)	.98	–0.0132 (0.780)	.99	0.113 (0.545)		.84	0.113 (0.615)	.85
	Race: Other	0.209 (0.631)	.74	0.209 (0.826)	.80	0.644 (0.614)		.30	0.644 (0.652)	.32
	Education: Bachelor’s degree	1.880 (0.600)	.003	1.880 (1.044)	.07	1.584 (0.495)		.002	1.584 (0.824)	.06
	Education: Master’s degree	1.586 (0.738)	.04	1.586 (1.128)	.16	1.014 (0.586)		.09	1.014 (0.890)	.25
	Education: PhD	0.380 (0.935)	.69	0.380 (1.245)	.76	–0.158 (0.764)		.84	–0.158 (0.982)	.87
	Occupational status: working full time	–0.345 (0.413)	.41	–0.345 (0.790)	.66	–0.128 (0.439)		.77	–0.128 (0.623)	.84
	Occupational status: working part time	0.332 (0.425)	.44	0.332 (0.801)	.68	0.326 (0.448)		.47	0.326 (0.632)	.61
	Occupational status: unemployed	–0.760 (0.485)	.12	–0.760 (1.031)	.46	–0.719 (0.698)		.31	–0.719 (0.813)	.38
	Urban	–0.451 (0.433)	.30	–0.451 (0.512)	.38	–0.472 (0.409)		.25	–0.472 (0.404)	.24

^a^Dependent variable: diagnostic performance.

^b^Dependent variable: clinical task performance.

^c^OLS: ordinary least squares.

^d^Robust standard errors are clustered by each participant.

^e^R^2^=0.347.

^f^57 groups (clusters).

^g^Variance structures were specified using unstructured, identify, and exchangeable, respectively, and results qualitatively remained the same.

^h^R^2^=0.412.

## Discussion

### Principal Findings

This mixed methods study aimed to explore the status of AI-assisted decision-making patterns among clinicians and gain a detailed understanding of how this novel method of AI-human collaboration and decision making could progress in the future. The results from the qualitative methods showed that clinicians described the diagnostic process with the support of AI as more positive compared with encountering a live patient on their own. Nonetheless, the respondents’ keywords revealed that AI assistance impeded clinicians from formulating their own subjective diagnoses based on their clinical reasoning and that the main complaints among clinicians related to some of the steps in the AI algorithms. Moreover, our quantitative results showed that clinicians’ perceptions of their clinical diagnostic capability neither indicated the current level of AI assistance nor enhanced their care task performance. The participants believed that their *ex ante* quality and capability, such as education, age, and daily technology habits, were more relevant in enhancing their care task performance.

### Comparison With Prior Work

We expected our research to make 2 important contributions to the existing IS and health care literature. First, our findings from clinicians’ keywords in their textual comments demonstrated that clinicians perceived AI assistance positively. However, the current AI interface may not align with their clinical reasoning process, and therefore, such AI interface issues can negatively affect clinicians’ perception of whether AI can collaborate with them as a team member. Clinician keywords such as “long system,” “strict sensitive clicking,” “differential diagnosis,” “large learning curve,” “technical difficulties,” and “hard system” demonstrated human-computer interaction (HCI) issues. In other words, this phenomenon can be linked to the topic of the user interface and explainable or understandable case scenarios in AI research. Studies on HCI have typically focused on the effect of technology on users by considering principles, guidelines, and strategies for designing and interacting with user interfaces [57]. The importance of the design aspect of AI (or AI from an HCI perspective) is more pronounced in health care because active involvement of stakeholders in the design stage can promote appropriation and sensemaking of the focal technology and increase the benefits of AI implementation [58,59]. Studies on machine learning–human interaction have emphasized “human-centeredness” in the use of AI such that humans and machines can integrate or work together as a team [60]. To do so, AI must be explainable, comprehensible, useful, and usable for clinicians in the use scenarios, both in practice and in simulation.

However, some challenges exist. First, the incorporation of fast-moving machine learning techniques into common user experience designs falls under restrictions in environment, law, and regulations in real-world scenarios [57]. Second, there are unintended consequences of AI-generated automated inferences and functions under uncertainty [61] so that, in cases where scenarios yield false positives or false negatives, AI-based decision making negatively affects clinicians’ care task performance and patient health outcomes [57]. From the perspective of clinicians, case scenarios or process logic may be the primary interface faced by clinicians under the current development of AI algorithms. However, we also showed that the complexity of AI algorithms may disrupt clinicians’ patient diagnostic decisions because clinicians may not understand how to access the complete functions and capabilities of the AI [36]. Thus, based on the previous literature and current evidence in this paper, our results call for more attention to HCI issues by actively involving the end users in the system development procedures and providing them with adequate education to co-create effective decision-making scenarios with AI assistance.

For another, we empirically quantified the effect of AI assistance on clinicians’ decision making in a nomological network and explored whether it can serve as a factor to enhance clinical diagnostic capability and overall health task performance. Our results demonstrated that, although clinicians interacted with AI algorithms in safer simulations, the effect of AI assistance was either negative or nonexistent in the clinicians’ diagnostic decisions. Instead, the clinicians’ *ex ante* personal traits, that is education and age, are positively associated with enhanced outcomes. This finding corresponds with the existing understanding that years of training, professional background, and educational background affect diagnostic performance [62]. Moreover, we found that AI assistance was negatively associated with the clinicians’ perceptions of task performance. This might be due to frontline health care providers’ trust in AI [14] or implementation issues [63] in health care. It will be worthwhile to revisit our research model to identify AI-specific factors and test downstream effects on clinicians’ AI use performance in future research.

Furthermore, we found that the clinicians’ *ex ante* technological traits were statistically associated with clinical decision making. First, we found that technological habits were positively linked to clinical diagnostic and health care task performance. In this study, we viewed a technological habit as a social media habit. Clinicians have used social media technologies that may embed AI algorithms and techniques in nonhealth care contexts [64]. It is likely that processing and accessing multimedia-based information daily may help clinicians manage multimedia-based patient information on AI platforms. Second, we showed that computer literacy was negatively associated with overall health care task performance with AI assistance. In health care, clinicians’ levels of literacy vary across contexts such as information [65], health [66], YouTube [67], informatics tools [68], and computers [69]. In particular, the concept of computer literacy is broad and encompasses hardware and software [69], informatics tools (eg, decision support systems), handheld devices [68], computerized statistical analysis, databases, presentation graphics, spreadsheet applications, and bibliographic database searches for evidence-based practice [70]. Such measures of literacy are related to knowledge about focal tools or interpreting health information in health care contexts. Meanwhile, a renewed concept of computer literacy has emerged, known as “digital literacy,” or the combined knowledge, skills, and competencies necessary for thriving in a technology-saturated culture [71]; it encompasses various forms of literacy via visual, electronic, and digital forms of expression and communication [72]. The use of 3-dimensional virtual images in operations or virtual reality goggles in pathology laboratories [73] or the matching of doctors with professional actors in medical improvisation sessions over Zoom [74] are adequate examples of computer literacy with which clinicians should be equipped. Therefore, it is necessary to redefine and contextualize the definition of computer literacy in the domain of AI use and actively educate clinicians on this topic in practice and in training altogether.

The findings of this study have various practical implications. With an explicit focus on the clinicians’ AI-assisted decision making, we found that their sensemaking of the diagnostic logic provided by AI and the system features may pose a challenge to the health care workforce. Our findings correspond with a recent report by the National Academy of Medicine [75] that highlighted “augmented intelligence,” with an emphasis on the supporting role of AI in data synthesis, interpretation, and decision making for health care multi-stakeholders, such as clinicians, patients, and other related professionals. In preparing clinicians for such a change, the report suggested that their training should incorporate education programs on how to assess and use AI products and services appropriately for new and incumbent professionals alike [75].

Moreover, to prevent AI algorithms from generating a simplified decision plan, health care providers should be involved in the case scenarios of patient diagnosis to enhance the effectiveness of the AI algorithms [36]. What is largely neglected in the discussion is the need to close gaps between practice and clinical training for increasing AI understanding. The developers of AI need to consider the clinicians’ current levels of exposure to technology in practice and in simulation and then design clinician-centered algorithms and interfaces. To achieve the goal of human-AI collaboration in health care [76], beyond involving clinicians in the development of clinical AI algorithms, these algorithms and their interfaces should be more human-centered. There must be assessments of diagnoses made by AI in both practice and training to reduce the gap between theory and practice in the use of AI by the health care workforce. This can be done by gathering behavioral big data from clinicians in various disciplines to help align AI algorithms with the unique, subjective patterns of reasoning that humans employ in clinical diagnosis.

### Limitations

As with all research, this study is not without limitations, and its results should be interpreted with caution. First, we employed purposive sampling techniques for the target population. Since our target audience was individuals with experience in clinical diagnosis in various scenarios, we targeted and recruited FNP students as our sample. As such, the response rate was relatively low, and missing values were prevalent in the data. Future research could benefit from increasing the sample size to compare group differences in greater depth. Although our sample represents the target population of this study, sampling at the national level would be beneficial for generalizing the results of this study. Second, our AI variable was operationalized as binary (1: AI assistance; 0: otherwise). Future research may consider a survey construct with items that capture the rich characteristics of multimedia technology variables in the research models. Lastly, as our results were derived from the use of AI-enabled diagnostic technology, our results may not be generalizable to other types of AI or other clinical decision-making categories.

### Conclusions

In keeping with the recent interest in and expectations for AI-assisted decision making in health care, as a first step, our research explored clinicians’ sentiments toward AI assistance and their perceptions using sentiment analysis and a mixed methods design. Our results indicated that, while there are negative or nonexistent effects of AI assistance in enhancing clinical decision making, clinicians have positive sentiments toward AI assistance in the decision-making process, comparable with their encounters with actual patients. With this potential, we suggest that health care leaders, policy makers, and AI developers need to collect clinicians’ behavior data and revisit the design and user interface of AI to make it more clinician-centered and collaborative.

## Abbreviations

AI: artificial intelligence
CTA: Consumer Technology Association
FNP: family nurse practitioner
HIT: health information technology
HLM: hierarchical linear modeling
IS: information systems
NLU: natural language understanding
